# Towards Using NMR to Screen for Spoiled Tomatoes Stored in 1,000 L, Aseptically Sealed, Metal-Lined Totes

**DOI:** 10.3390/s140304167

**Published:** 2014-03-03

**Authors:** Michael D. Pinter, Tod Harter, Michael J. McCarthy, Matthew P. Augustine

**Affiliations:** 1 Department of Chemistry, University of California, One Shields Avenue, Davis, CA 95616, USA; mdpinter@ucdavis.edu; 2 The Morning Star Packing Company, Woodland, CA 95695, USA; tharter@morningstarco.com; 3 Department of Food Science and Technology, University of California, One Shields Avenue, Davis, CA 95616, USA; mjmccarthy@ucdavis.edu

**Keywords:** nuclear magnetic resonance, tomato paste, spoilage, single-sided coil, T_1_ relaxation, saturation recovery, metal container

## Abstract

Nuclear magnetic resonance (NMR) spectroscopy is used to track factory relevant tomato paste spoilage. It was found that spoilage in tomato paste test samples leads to longer spin lattice relaxation times T_1_ using a conventional low magnetic field NMR system. The increase in T_1_ value for contaminated samples over a five day room temperature exposure period prompted the work to be extended to the study of industry standard, 1,000 L, non-ferrous, metal-lined totes. NMR signals and T_1_ values were recovered from a large format container with a single-sided NMR sensor. The results of this work suggest that a handheld NMR device can be used to study tomato paste spoilage in factory process environments.

## 1. Introduction

Globally, around 1.3 billion tons of food is wasted per year. In North America alone, approximately 175 kg food/person is compromised at the pre-consumption stages of production [1]. In order to minimize this loss, food packaging companies are constantly seeking ways to improve packing process sanitation. In spite of these efforts, spoilage is never entirely eliminated. The direct consequence of a few spoiled containers translates into four to six figure financial losses when both the spoiled product and waste disposal are considered [1]. Bacterial contamination is a primary source of food spoilage that many industrial food processes attempt to eliminate by operating in aseptic environments [2]. Once introduced into a system, bacteria proliferate as a function of temperature and available metabolic nutrients. Technologies that provide early detection of bacterial growth can ultimately provide food companies with a competitive edge in the industry and decrease the environmental impact of waste disposal.

The tomato paste processing industry is interested in spoilage because contaminated tomato paste spoils in a matter of days. Industrial tomato paste totes or bags that comprise an inner plastic sack and a thin outer aluminum layer are designed to accommodate a 1,000 L volume [3], which means that the introduction of even a small amount of contamination is costly. The tomato paste industry is interested in developing ways to detect tomato spoilage in these 1,000 L non-ferrous, metal-lined containers without violating the seal. Although early spoilage detection cannot preserve the contents of the compromised tote, it would eliminate costs associated with shipping, disposal, and frustration incurred when a tote with spoiled tomato paste arrives at its destination.

Several analytical methods that are traditionally used to measure food spoilage fail in the industrial tomato paste manufacturing domain. Laser and other optical methods that involve passing light through a sample require non-metal, transparent containers [4]. Mass spectrometry threatens tomato paste sterility as it is an invasive process that requires the container seal to be broken for sampling [4]. Nuclear magnetic resonance (NMR), on the other hand, is a non-invasive approach that has gained traction recently in studying problems in industrial process environments [5–7]. These examples share the common theme of being modifications to the conventional NMR approach involving a sample in a non-metal container enclosed by a large magnet. In the case of tomato paste these conditions do not apply. Firstly, the container is a 1,000 L non-ferrous, metal-lined tote that cannot fit inside of a standard magnetic resonance or imaging magnet. Moreover, the tote is filled with ca. 1 metric tonne of tomato paste and cannot be moved to the sensor. The aluminum lining in these totes presents an additional problem as eddy currents are generated on the surface of the metal that attenuate the applied radio frequency (rf) magnetic field [8]. The skin depth δ = (2/μσω)^1/2^, written in terms of the metal conductivity σ, the metal magnetic permeability μ, and angular frequency ω, is the distance into metal at which incident radiation is attenuated by *e*^−1^ [8]. Traditional NMR spectroscopy operates at Larmor frequencies ω_0_/2π = γ B_0_ > 100 MHz yielding δ < 8 μm for aluminum. This shielding prevents signals from being observed through the 75 μm thick aluminum layer incorporated into the tote material.

A useful food spoilage detector must have four essential design features. It should be noninvasive, portable, accept a variety of sample geometries, and probe both metal and non-metal containers. These features are provided by a unilateral NMR circuit, or single-sided coil, which is an NMR tank circuit with a planar inductor that can non-invasively probe a wide range of sample geometries [9]. Interfacing this coil with a light weight permanent magnet that produces a small static magnetic field ultimately leads to increased metal transparency. The decreased field strength leads to a lower Larmor frequency ω_0_/2π ≈ 5 MHz that concomitantly increases the skin depth in aluminum to δ ≈ 40 μm thus providing access to the study of the contents of non-ferrous, metal-lined containers. In practice, a portable single sided coil and magnet system could be placed flush with a 1,000 L industrial tomato tote to non-invasively provide spoilage information about the contents. Small, portable single sided coil and magnet systems are commercially available with the most well-known example being the NMR Mobile Universal Surface Explorer, developed at RWTH-Aachen [10].

The ultimate goal of this work was to determine if NMR spectroscopy is sensitive to tomato paste spoilage and to record the NMR signal from tomato paste in industrial, non-ferrous, metal-lined 1,000 L totes. To this end, tomato paste NMR relaxation parameters were monitored in small 100 mL glass containers using a customized low magnetic field NMR spectrometer to find a measureable variable that correlates with tomato spoilage. After the spoilage dependent NMR parameter was determined in non-metal containers, it was confirmed that it can be measured using a sample in a non-ferrous, metal-lined tote using a single sided coil/magnet system. As NMR experiments in metal containers are rarely reported in the literature, effort is spent in the next section describing the ω_0_/2π = 4 MHz electromagnet based and the ω_0_/2π = 5.25 MHz single sided magnet based systems.

## 2. Materials and Methods

Commercial tomato paste was used as received from The Morning Star Company. Small format samples were prepared in a clean room by aseptically transferring fresh tomato paste into thirty 100 mL VWR media bottles. The thirty identical samples were separated into two, separate, fifteen bottle sets. The control group was set aside while the other fifteen bottles were inoculated with a mixed culture of microorganisms that were harvested from a leaking aseptic filler line at the Los Ba ños, CA, Morning Star Company food packing facility. Immediately after preparation, the thirty samples were cooled to T < 5 °C and stored in a refrigerator. These samples were transported from The Morning Star Company to UC Davis in a standard cooler and were stored in the laboratory refrigerator until needed. Large format samples were contained in standard 1,000 L storage totes with a 75 μm thick metal lining. These aluminum layered, plastic bags were short filled with tomato paste, stored at room temperature, and shipped from The Morning Star Company to UC Davis.

**Figure 1.**
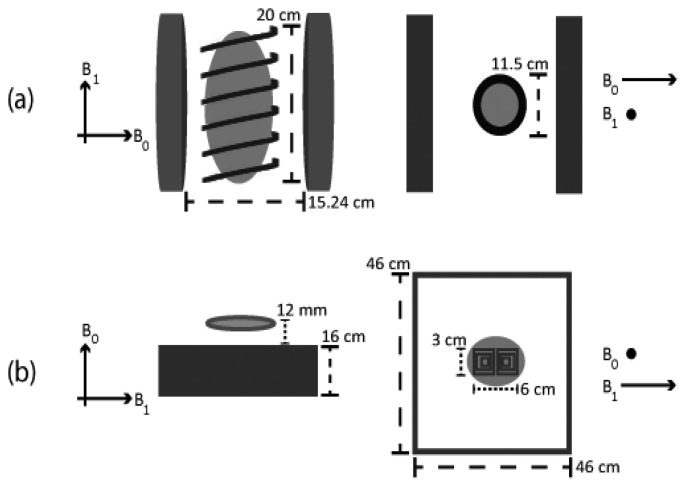
Diagrams comparing the conventional low field NMR instrument in (**a**) to the single sided instrument in (**b**). The primary difference between these two geometries is that applied rf interacts with the entire sample in (a) and only a fraction of the sample in (b) as shown by the shaded gray area.

A Tecmag Redstone NMR spectrometer was used to acquire all transient NMR relaxation signals from the small format 100 mL samples. As shown in Figure 1a, the small format samples were completely enclosed by the ω_0_/2π = 4 MHz tuned and matched, 11.5 cm diameter, 20 cm long, 11 turn variable pitch inductor situated inside of a homebuilt NMR probe. This probe was mounted inside of a B_0_ = 980 G SMIS electromagnet with a 15.24 cm pole face separation. A standard inversion recovery pulse sequence involving 625 W, 20 μs π/2 rf pulses and 21 non-linear sampled recovery times between 0 and 300 ms was used to recover T_1_ values while a multiple π rf pulse Carr-Purcell-Meiboom-Gill (CPMG) pulse sequence with an 8 ms π rf pulse separation involving the same rf pulse power and length was used to recover T_2_ values by non-linearly increasing the number of π rf pulses to 60 in 18 steps. All measurements reported for this geometry did not require signal averaging and corresponded to one transient signal per relaxation time point.

**Figure 2.**
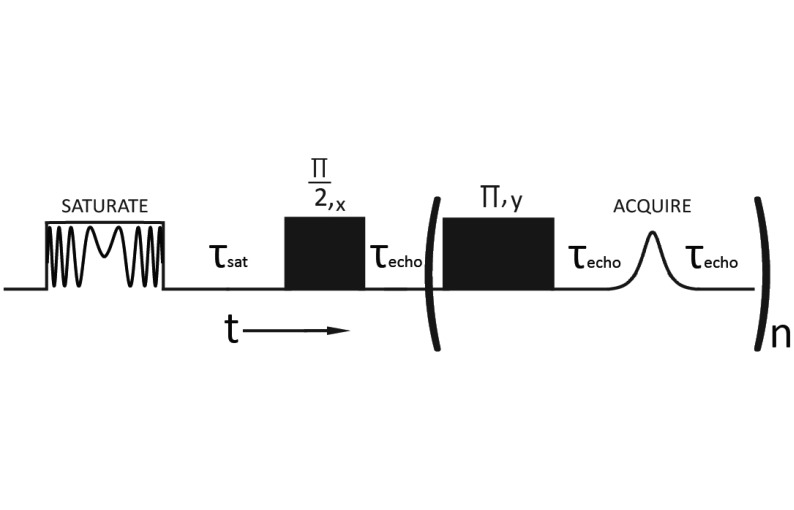
Modified saturation recovery pulse sequence that incorporates a ±75 kHz frequency sweep into the saturation pulse. The CPMG backend is required to observe a signal because of the large inhomogeneous static field and rf circuit ring down. The variable frequency rf pulse more efficiently saturates the magnetization in single sided mode in comparison to the conventional rf pulse train used for sample-in-the-coil geometries.

In the case of the large format metal-lined totes, the Tecmag Redstone NMR spectrometer was connected to an ABQMR single sided permanent magnet system. In this case the sample was not inside of the rf coil as shown in Figure 1b and the homogeneous sample volume of the B_0_ = 1,300 G static magnetic field was adjusted to be 12 mm above the surface of a flat rf coil tuned to ω_0_/2π = 5.25 MHz and designed to provide an ≈1 cm thick homogeneous rf excitation slice spatially coincident with the static magnetic field. The saturation recovery pulse sequence shown in Figure 2 used a 500 ms long, 25 W, ±75 kHz frequency swept rf pulse to saturate the wide NMR line presented by the single sided magnet and a CPMG spin echo train with 3 μs long, 625 W π/2 rf pulses, τ_echo_ = 1.5 ms and 500 μs< τ_sat_ < 3 s to observe the time domain signal. The sum of the first ten echoes in the CPMG spin echo train was used to estimate the signal intensity for T_1_ estimates. All measurements reported for this geometry required 30 transient signals to be averaged per relaxation time point.

Unless otherwise specified, all reported T_1_ and T_2_ values correspond to an average of the exponential signal decay constant over 15 samples while the 95% confidence limit was established using the standard deviation of the same data along with the appropriate Student t factor. All data processing was accomplished using Matlab.

## 3. Results

The 980 G SMIS electromagnet was used to obtain T_1_ and T_2_ values for all thirty samples as a function of time at both low and room temperature. The available room temperature shims in the electromagnet were used to reduce the NMR line width for these samples in the as received glass jars to less than 100 Hz.

**Figure 3.**
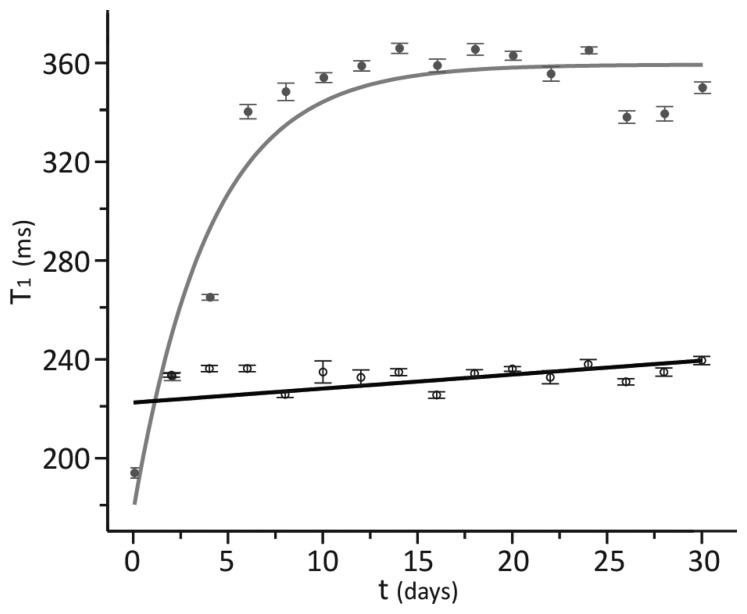
Comparison of sterile (open circles) to unsterile (solid circles) T_1_ values as a function of room temperature exposure time. The 95% confidence intervals corresponding to averaging over 15 samples are included in the plot.

The low temperature measurements were accomplished on ten randomly selected sterile and unsterile samples. These samples were only removed from the low temperature storage refrigerator for t ≈ 5 min to obtain the T_1_ and T_2_ values reported in Table 1. Given the heat capacity of the tomatoes and the glass container, the temperature rise during this time was less than +3 °C. As long as the storage temperature was maintained below 5 °C, the T_1_ and T_2_ values remain constant over the course of at least one month. Room temperature T_1_ and T_2_ measurements were accomplished on all thirty samples. The fifteen sterile and unsterile samples were separated into three separate batches. The first batch of five samples each was removed from the refrigerator and allowed to warm to room temperature over the course of an hour. The T_1_ and T_2_ values for these samples were monitored every two days for a thirty day period. At day fifteen of these measurements, a second batch of five samples each was removed from the refrigerator and the measurements were repeated in parallel with the first batch. On the final day of measurements for the first batch, the third batch of five samples each was removed from the refrigerator and measurements were repeated in parallel with the second batch. Although the room temperature T_2_ value was constant as a function of time for these samples as shown in Table 1, the corresponding T_1_ values as shown in Figure 3 were not.

**Figure 4.**
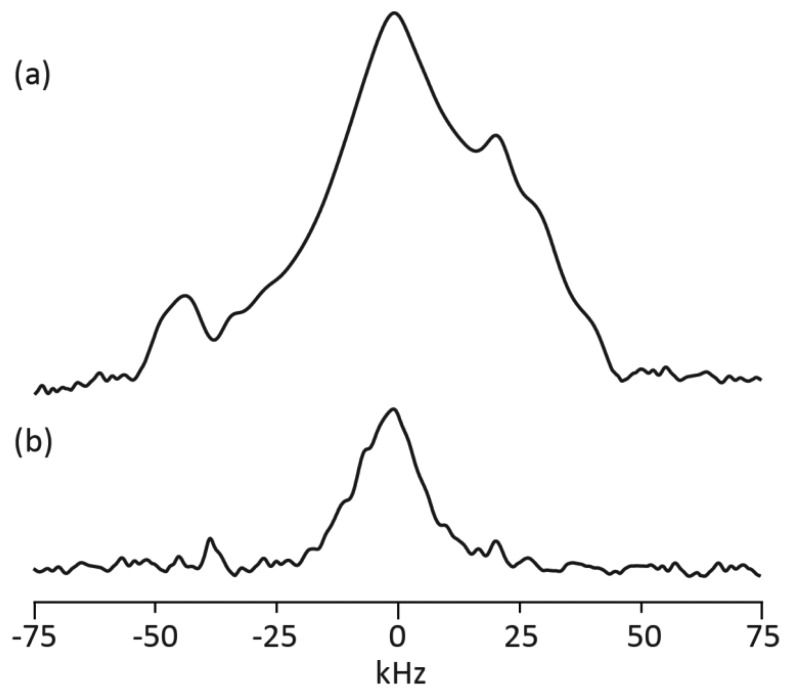
Example ^1^H NMR spectra obtained with the single sided coil magnet for a 100 mL tomato paste sample without (**a**) and enclosed in (**b**) the aluminum lined tote material.

Regression of these T_1_ measurements to an exponentially rising function yields the steady state spin lattice relaxation time T_1_∞ and the time constant for this increase τ, which are provided in Table 1. The lack of room temperature shims in the 1,300 G single sided magnet provided a wide, inhomogeneously broadened NMR peak as shown in Figure 4a for tomato paste in a 100 mL VWR glass jar. The consequence of the metal-lined tote on this signal is demonstrated in Figure 4b for the same glass jar wrapped in the aseptic, metal-lined tote material. The ability to measure a signal from a 100 mL tomato sample wrapped with the tote material in Figure 4b prompted the recovery of the T_1_= 226 ± 28 ms value for tomato paste short filled into a 1,000 L, aseptic, metal-lined tote.

## 4. Discussion

The goal of this work was to determine if NMR spectroscopy could be used to identify spoiled tomato paste stored in industry standard, 1,000 L, large format, non-ferrous, metal-lined totes. There are three challenges that were met in order to accomplish this goal. Two of these, the identification of an NMR parameter that reflects tomato paste spoilage and the recovery of an NMR signal from a sample enclosed in a non-ferrous, metallic shield, were addressed using the standard NMR geometry provided by the 980 G electromagnet. Finally, the single sided system was used to measure the T_1_ relaxation time for tomato paste in a short-filled, 1,000 L, non-ferrous, metal-lined tote.

In order to address the first of these challenges, a set of known sterile and unsterile tomato paste samples was studied in ideal laboratory conditions using a conventional NMR magnet that surrounds the small format samples. The results, shown in Table 1, report virtually no variability (ca. 18 ms) in the T_1_ and T_2_ values between the as received sterile and unsterile samples at low temperature. This result suggests that the low temperature either halts or significantly slows the bacteria mediated tomato paste spoilage. At room temperature where tomato processing, packaging, and storage occur, the relaxation times shown in Table 1 for sterile and unsterile samples are different. The ca. 115 ms difference in T_1_^∞^ values can be used to identify spoiled tomato paste. As shown in Figure 3, the steady state T_1_^∞^ value difference between sterile and unsterile samples takes ca. 5 days to develop at room temperature reflecting the rate of bacteria growth in the closed sample.

The mechanism of the relationship between bacteria mediated tomato paste spoilage and changes in T_1_^∞^ was not explored in detail in this work. An obvious possibility for this dependence is that the bacterial metabolism either directly or indirectly chemically changes the tomato substrate. In the direct process, bacteria directly consume the substrate, while in the indirect case, the products of bacterial metabolism react with the tomato substrate or simply dilute the mixture, decreasing the sample viscosity. The decreased sample viscosity translates into a larger T_1_^∞^ value [11]. It is likely that the mechanism depends on the bacteria identity and current work involves exploring how the available NMR parameter values change with different spoilage agents. From the point of view of this work, all that matters is that the T_1_^∞^ value for unsterile samples measurably deviates from the sterile control set value.

Operation at a decreased magnetic field strength in comparison to standard NMR spectroscopy was motivated by the need to recover signals from samples enclosed in non-ferrous, metallic containers. Here the lower induced Larmor frequency ca. 4–5 MHz increases the penetration depth of the applied and detected radio frequency fields by about an order of magnitude in comparison to that anticipated by using typical 400–500 MHz values. As long as the effect of the non-ferrous metallic container on the tank circuit tuning and impedance matching properties have been properly compensated, the metal container can be modeled as a simple attenuator. In other words, the 4 MHz resonance frequency of the NMR tank circuit shifts to higher frequency when a non-ferrous, metallic container is studied. In the case of the plastic coated, 75 μm thick aluminum aseptic tote material, the shift is ca. 1 MHz. The addition of extra capacitance in parallel with both the tuning and matching capacitors returns the circuit resonance frequency to the original 4 MHz value. Although the circuit quality factor after this adjustment returns to the metal free value, the optimum π/2 rf pulse length is longer for the same applied rf power and the signal size is smaller. Similar efforts were necessary when the unique single sided coil/magnet system were used. The increased rf pulse length and decreased signal size can be appreciated with reference to Figure 4. Here the NMR signals for a 100 mL VWR bottle without and with the aseptic tote material are compared in Figure 4a,b, respectively. The decreased excitation bandwidth and signal-to-noise ratio in Figure 4b in comparison to Figure 4a demonstrate the attenuation effect.

The homogeneity of the applied static magnetic field in the single sided geometry is significantly less than in the standard geometry. For example, the ^1^H line widths for the same 100 mL VWR sample are ca. 100 Hz and 50 kHz for the electromagnet and the single sided magnet respectively. The increased line width in the single sided geometry combined with the decreased excitation bandwidth shown in Figure 4 impacts rf pulse design. Specifically, the standard inversion recovery measurement of the T_1_ relaxation time constant does not work in the single sided geometry when non-ferrous, metal containers are used because the signal cannot be completely inverted. Moreover, the inhomogeneous spectral line width is so large that the signal decays during the rf pulse ring down period preventing transient signal detection. The pulse sequence shown in Figure 2 solves both of these problems. Instead of inverting the magnetization M_0_, a swept frequency saturation pulse is used. In this way the recovery of magnetization from 0 to +M_0_ is monitored in the saturation recovery pulse sequence instead of the usual −M_0_ to +M_0_ transformation used in the inversion recovery experiment. The signal damping issue is also resolved by this pulse sequence since a spin echo with a pulse delay greater than the radio frequency ring down time is used. An added benefit of the saturation recovery approach shown in Figure 2 is overall experiment time. Since the signal is recovering from a saturated state, the usual 5 T_1_ waiting period between data acquisition events required for the inversion recovery pulse sequence is not necessary.

As a final test of the ability of NMR spectroscopy to study tomato paste spoilage in a large format, 1,000 L, non-ferrous, metal-lined tote, the T_1_ = 226 ± 28 ms relaxation time constant was obtained from a factory sealed short filled tote using the single sided magnet and the pulse sequence shown in Figure 2. The order of magnitude greater error recognized in the single sided T_1_ measurement in comparison to the error for the standard geometry is not problematic as the increase in T_1_ in the unsterile case is four to five times greater than this uncertainty. Improvements on the single sided T_1_ error can also be made by thermally stabilizing the single sided magnet. The experiments reported here operate the ABQMR system in a standard laboratory environment subject to uncontrolled temperature variations. Indeed on the basis of the room temperature electromagnet T_1_ values shown in Table 1, the 226 ± 28 ms T_1_ value for the short filled 1,000 L tote obtained from the single sided magnet suggests that the tomato paste in the detection volume near the tote surface is sterile and thus suitable for distribution and human consumption. Extending this NMR based local measurement to the entire contents of the 1,000 L tote is straightforward given that existing observations of spoilage are made by physically viewing the surface condition of tote stored paste. Since tomato concentrate is a liquid based product significant spoilage impacts the vast majority of the material in a tote. The concern for processors is not minor pockets of local bacteria growth but significant proliferation that impacts the bulk properties of the entire tomato concentrate and hence one or several local surface measurements is adequate to estimate spoilage.

## 5. Conclusions

The purpose of this work was to demonstrate that NMR spectroscopy could be used to study tomato paste spoilage in 1,000 L, large format, non-ferrous, metal-lined totes. To accomplish this goal, a reduced magnetic field was used to explore the relaxation properties of sterile and unsterile samples. It was found that the increase in T_1_ values for the unsterile samples could be used as a metric for spoilage and that the reduced operating frequency permitted measurement of the T_1_ values for standard samples enclosed in aseptic metal-lined totes. Introduction of a single sided magnet afforded the measurement of T_1_ for tomato paste in a large format tote for the first time and, coupled with the small sample T_1_ metrics, can be used to non-invasively monitor tomato paste spoilage.

Two extensions of this work are being considered. The unsterile tomato paste sample set used in this study was generated from a realistic factory contamination source. One extension currently underway involves a more thorough investigation of the relationship between measured T_1_ values and tomato spoilage mechanisms. Contamination sources include real in-the-field locations as well as documented bacterial strains. The second extension involves downsizing the single sided ABQMR magnet system to a hand-held NMR MOUSE device [10]. It is this portable instrument that will be useful for screening filled large format totes in a factory process environment.

## Figures and Tables

**Table 1.** NMR Relaxation Parameters for Tomato Samples.

	**T <5°C**		**T > 25 °C**		

	**T_1_(ms)**	**T_2_(ms)**	**T_1_^∞^ (ms)**	**τ (days)**	**T_2_ (ms)**
Sterile	217.3 ± 2.2	53.5 ± 1.4	234.8 ± 1.8	-	58.8 ± 1.0
Unsterile	199.4 ± 1.3	47.2 ± 0.9	349.0 ± 2.6	4.0 ± 1.8	57.0 ± 0.5
